# Therapeutic Targeting Hypoxia-Inducible Factor (HIF-1) in Cancer: Cutting Gordian Knot of Cancer Cell Metabolism

**DOI:** 10.3389/fgene.2022.849040

**Published:** 2022-03-31

**Authors:** Abhilasha Sharma, Sonam Sinha, Neeta Shrivastava

**Affiliations:** ^1^ Department of Life Science, University School of Sciences, Gujarat University, Ahmedabad, India; ^2^ Kashiv Biosciences, Ahmedabad, India; ^3^ Shri B.V. Patel Education Trust, Ahmedabad, India

**Keywords:** genomic alterations, cancer, metabolism, warburg effect, hypoxia-induced tumor microenvironment, metabolic reprogramming, cancer therapies, clinical outcomes

## Abstract

Metabolic alterations are one of the hallmarks of cancer, which has recently gained great attention. Increased glucose absorption and lactate secretion in cancer cells are characterized by the Warburg effect, which is caused by the metabolic changes in the tumor tissue. Cancer cells switch from oxidative phosphorylation (OXPHOS) to aerobic glycolysis due to changes in glucose degradation mechanisms, a process known as “metabolic reprogramming”. As a result, proteins involved in mediating the altered metabolic pathways identified in cancer cells pose novel therapeutic targets. Hypoxic tumor microenvironment (HTM) is anticipated to trigger and promote metabolic alterations, oncogene activation, epithelial-mesenchymal transition, and drug resistance, all of which are hallmarks of aggressive cancer behaviour. Angiogenesis, erythropoiesis, glycolysis regulation, glucose transport, acidosis regulators have all been orchestrated through the activation and stability of a transcription factor termed hypoxia-inducible factor-1 (HIF-1), hence altering crucial Warburg effect activities. Therefore, targeting HIF-1 as a cancer therapy seems like an extremely rational approach as it is directly involved in the shift of cancer tissue. In this mini-review, we present a brief overview of the function of HIF-1 in hypoxic glycolysis with a particular focus on novel therapeutic strategies currently available.

## Introduction

Increased incidence of cancer patients around the globe clearly alarms for more comprehensive research of this life-threatening problem. The initiation of cancer is a multi-step process that includes genomic alterations. Hannah and Weinberg have extensively described the “hallmarks of cancer”, one of which is “metabolic reprogramming” that has recently emerged as a core trait of tumors (Hanahan and Weinberg, 2011; Hanahan, 2022). Specifically, the altered glycolytic metabolism pathway results in switching from oxidative phosphorylation (OXPHOS) in the mitochondria to aerobic glycolysis even in the abundance of oxygen in various cancer types. The “Warburg effect”, proposed by Otto Warburg over a century ago, was the first to reveal basic metabolic distinctions between differentiated cells and rapidly proliferating tumor cells (Otto, 2016). Warburg effect is the result of the interplay between (normoxic/hypoxic) HIF-1 upregulation, activation of an oncogene (cMyc, Ras), loss of function of tumor suppressors (mutant-p53, mutant-PTEN, micro RNAs and sirtuins with suppressor functions), activation of (PI3K/Akt/mTOR; Ras/Raf/Mek/Erk/cMyc; Jak/Stat3) or deactivation of (LKb1/AMPk) signalling pathways (Arora et al., 2015; Vaupel and Multhoff, 2021). Although Warburg’s and others’ findings have had a significant impact on our understanding of tumor biology, they constitute only one aspect of tumor metabolism.

In fact, cancer metabolism alterations span a wide range of metabolic pathways that serve a multitude of functions such as apoptosis, angiogenesis, anti-anoikis, and anchorage-independent expansion in cancer cells and in the tumor microenvironment (TME), in addition to glucose metabolism and energetics (Casero and Pegg, 2009; Platten et al., 2012; Zhang and Du, 2012; Jeon and Hay, 2018). Therefore, targeting the energy metabolism of cancer cells, which takes advantage of the metabolic differences between cancer cells and normal cells opens the doorway to novel therapeutic interventions.

The TME endures biochemical alterations during the growth of the solid tumor, including depletion of glucose, bicarbonate, and oxygen (i.e., hypoxia and anoxia), high amounts of lactate and adenosine, and low pH value (Wang et al., 1995; Ke and Costa, 2006). Hypoxia, a prevalent characteristic of cancer especially solid tumors, is hypothesised to enhance tumor invasiveness and metastasis (Ke and Costa, 2006). Tumor hypoxia has been attributed to a variety of factors. First, angiogenesis inability to keep up with cancer growth, such as the need for the cancer cell mass “outstripping” the ability of blood vessels to carry oxygenated blood. Second, ischemia-induced by arteriovenous shunting or microvessel ‘steal’ syndromes induced by abnormal vessel arborization and aberrant vascular connections inside malignancies. Lastly, elevated hydrostatic pressure within the tumor, results in compression of the microvasculature (Heldin et al., 2004). Several mechanisms, notably the hypoxia-inducible factor-1 (HIF-1) pathway, which promotes the elevated expression of glycolytic enzymes, can govern the metabolic transition state above at the transcriptional level. As a result, tumor hypoxia and HIFs influence the majority of cancer “hallmarks”, including cellular proliferation, apoptosis, metabolism, immunological responses, genomic instability, vascularization, neovascularization, invasion, and metastasis (Wigerup et al., 2016). Moreover, HIFs seem to impact chemo and radiation resistance through multiple pathways. Additionally, HIFs expression has been linked to poor prognosis and treatment relapse in clinical tumor samples (Sørensen and Horsman, 2020). Thus, HIFs appear to be critical therapeutic targets that can be used to enhance current cancer treatment for metastatic and treatment-resistant cancers.

The primary intent of this mini-review is to provide a brief overview of the metabolic processes that are regulated by a hypoxia-inducible factor. In this review, we outline the relevance of HIFs in glycolysis, cancer progression and the epithelial-mesenchymal transition (EMT). A further goal of the review is to overview the currently available therapeutic strategies.

### Relevance of HIF-1 Stimulated Glycolysis in Hypoxia

Hypoxia affects metabolic pathways in a variety of ways. For example, by blocking the oxygen-dependent process of mitochondrial OXPHOS, hypoxia reduces ATP synthesis, and thus makes O_2_-independent glycolysis a more important energy source (Denko, 2008; Frezza and Gottlieb, 2009). Increased glycolysis generates ATP quickly, but at the price of a substantial amount of glucose, as seen by elevated lactic acid levels. Intra-tumoral acidosis is mediated by the latter, in conjugation with mitochondria’s impaired capacity to use protons in ATP synthesis (Zhou et al., 2006). Surprisingly, rather than being anti-cancer, the stress placed on cancer cells appears to promote the formation of more aggressive subclones with a greater ability to penetrate tissues and metastasis (Gatenby and Gillies, 2004; Gatenby et al., 2007). Hypoxia-induced events are mostly determined by the activity of the transcriptional regulators’ hypoxia-inducible factor-1α (HIF-1α) and its partner HIF-1β.

**FIGURE 1:**
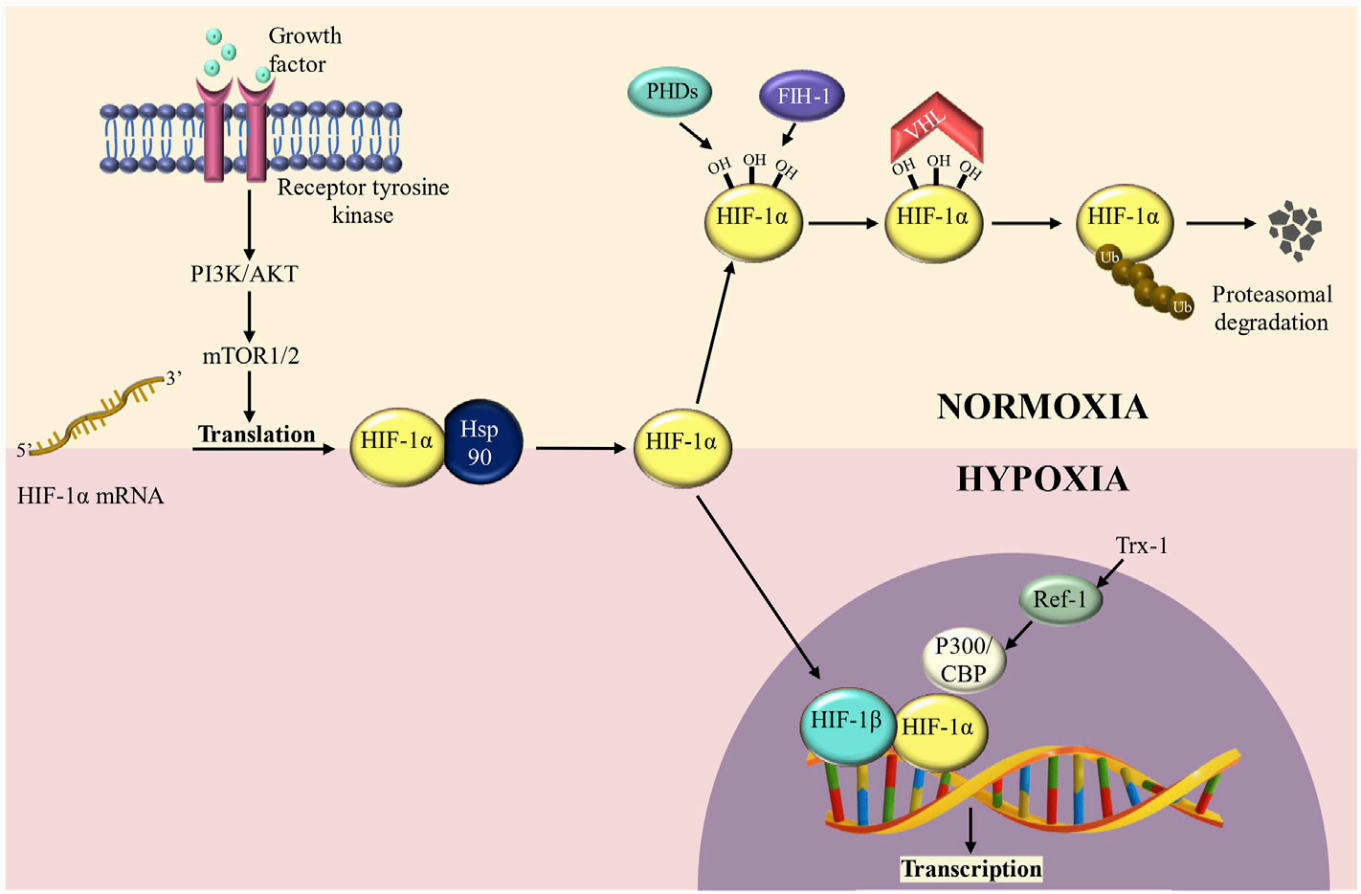
HIF-1α regulation in normoxic and hypoxic conditions. HIF-1α is hydroxylated at conserved residue (Proline 564**)** under normoxic conditions, a process mediated by prolyl-4- hydroxylases (PHDs) and factor inhibiting HIF-1 (FIH-1) enzymes. PHD hydroxylation promotes HIF-1α protein destabilization, whereas FIH-1 hydroxylation inhibits transcriptional activity by preventing interaction with CBP/p300. HIF-degradation is mediated by a ubiquitin-dependent process carried out by the Von Hippel-Lindau (VHL) E3 ubiquitin ligase complex. Under hypoxic circumstances, inactivation of PHDs and FIH-1 causes HIF-stabilization, followed by translocation into the nucleus and dimerization with HIF-1/ARNT to create the HIF transcription factor. During hypoxia, HIFs, in collaboration with the coactivator CBP/p300, promote transcription of a wide range of target genes.

HIF-1, a transcription factor, regulates the activation of several genes involved in glucose uptake and metabolism, cell survival/proliferation, angiogenesis, invasion, and metastasis (Semenza et al., 1994; Carmeliet et al., 1998). It is a heterodimer of HIF-1α and a constitutively expressed subunit HIF-1β which also forms a dimer with HIF-2α and regulates gene activation (Wang et al., 1995; Carmeliet et al., 1998). HIF-1α is generally targeted for ubiquitin-mediated destruction by proline hydroxylation and association with the Von Hippel-Lindau (VHL) tumor suppressor complex under normoxic conditions, but it is stabilised when the partial pressure of oxygen is low **(** Figure 1 **)**. Moreover, overexpression of HIF-1α is linked to a poor prognosis in various patients with human malignancies including breast, colon, gastric, lung, skin, ovarian, pancreatic, prostate, and renal cancer (Bos et al., 2001; Dales et al., 2005; Chen et al., 2007; Simiantonaki et al., 2008). Thus, HIF-1α significantly enhances our molecular understanding of cancer progression and metastasis which is discussed in detail in the following sections.

### Hypoxic Tumor-Microenvironment: Leading to Cancer Progression and Epithelial-Mesenchymal Transition

Mammalian cancer cells within a Hypoxic tumor microenvironment (HTM) undergo tremendous alterations, eventually intensifying their malignant activity. As a result, emphasis has been laid on identifying processes involved in cancer cell adaptation to the HTM in order to identify targets for potential therapeutic treatments (Liu et al., 2011; Kogita et al., 2014; Yang et al., 2015). Basically, in hypoxia conditions, HIF-1α forms the HIF complex, which functions as a transcription factor in the activation of a wide range of genes, orchestrating major phenotypic alterations and eventually leading to EMT. Following EMT, cells lose their normal morphology and gain mesenchymal traits (Kalluri and Weinberg, 2009; Singh and Settleman, 2010), including the development of stemness (Sutherland, 1988), increased invasiveness, and metastasizing capacities (Vaupel, 2004). All of these alterations have been associated with poor prognosis and chemotherapy resistance in a variety of tumor types (Yang et al., 2008; Chou et al., 2012). EMT is characterised by the loss of cell adhesion protein (for instance E-cadherin) and the elevated expression of mesenchymal-specific proteins such as SNAIL, Vimentin, and TWIST. As a matter of fact, this phenotypic shift has been highlighted as a major phase in the intricate process of developing distant metastasis (Chaffer and Weinberg, 2011; Valastyan and Weinberg, 2011).

**FIGURE 2:**
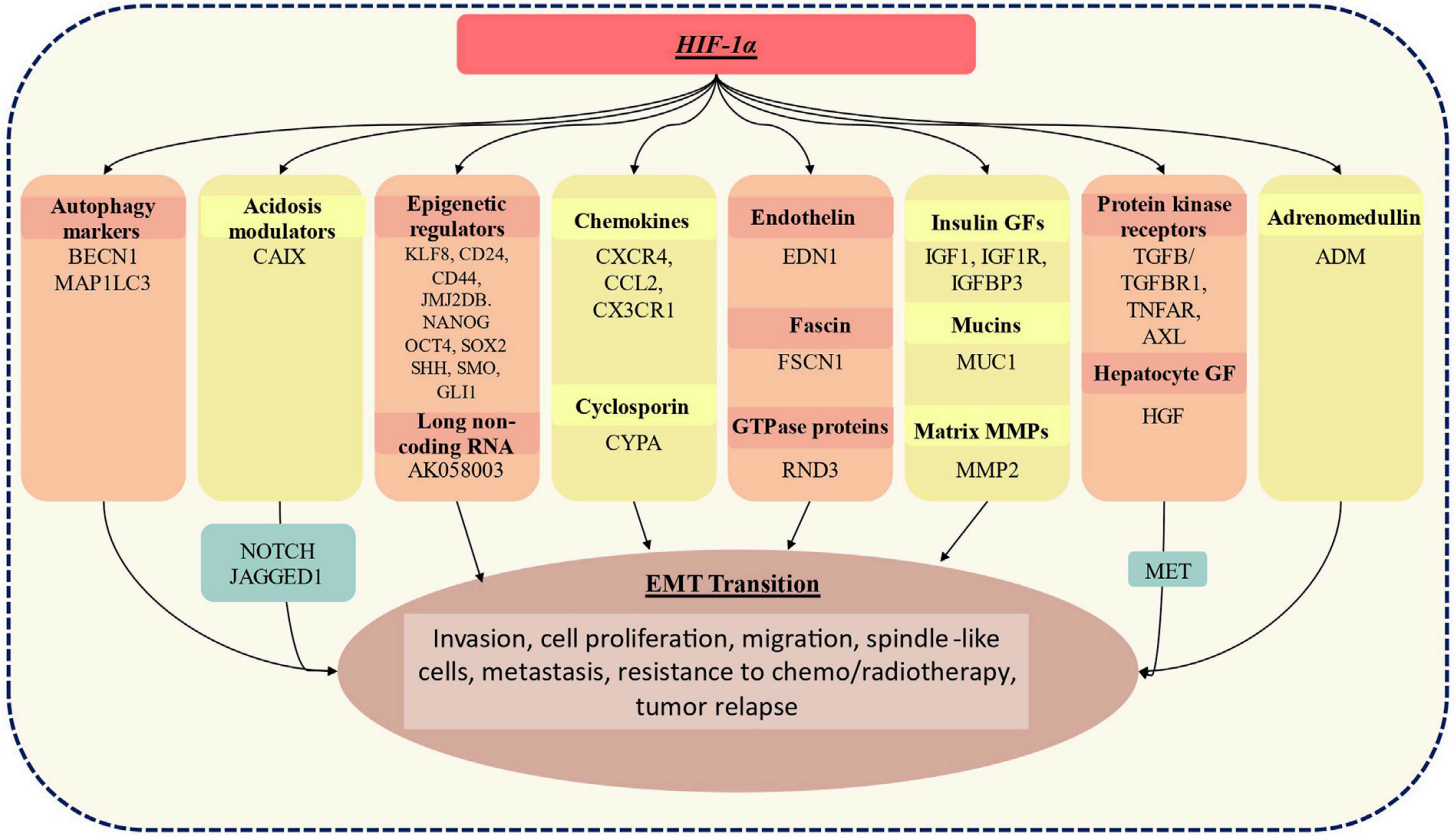
Genes whose expression has been linked to the activation status of HIF-1α, resulting in EMT. HIF-1α induces expression of BECN1, MAP1lC3 which is an autophagy marker; CAIX, acidosis modulators: epigenetic regulators: KLF8, cell surface glycoproteins (CD24, CD44), JMJ2DB which is lysine-specific demethylase jumonji domain, Nanog homeobox (NANOG), Octamer-binding transcription factor 4(OCT4), SRY sex-determining region Y-box (SOX2), sonic hedgehog (SHH), smoothened frizzled class receptor (SMO), GLI family zinc finger 1 (GLI1); AK058003- long non-coding RNA; multiple chemokines: CXCR4, CCL2, CCR7, CX3CR1; cyclosporin bind protein cyclophilin A (CYPA); endothelins: EDN1 (endothelin1; fascins: fascin actin-bundling protein 1(FSCN1); GTPase proteins: Rho family GTPase 3 (RND3): insulin growth factor which includes IGF1, IGF1R, IGFBP3; mucin 1, cell surface-associated (MUC1); matrix metalloproteinase; MMP2, protein kinases receptors including TGFb/TGFBR1, TNFAR, AXL; hepatocyte growth factor (HGF) which is a ligand of MET tyrosine kinase receptor; adrenomedullin (ADM). These activated genes are known to play a crucial role in EMT transition and result in increased invasiveness, cellular proliferation, migration, spindle-like cellular appearance, resistance to chemo/radiotherapy and tumor relapse.

As represented in Figure 2, the HIF-1α complex activates a number of key genes that mediate hypoxia > HIF > EMT axis. This axis has been extensively investigated in many aggressive tumors including lung, triple-negative breast cancer (TNBC), pancreatic ductal adenocarcinoma (PDAC) and renal cell carcinoma (RCC). For instance, autophagy markers (BECN1 and MAP1LC3) are activated in lung and PDAC (Zhu et al., 2014; Zou et al., 2014); overexpression of CAIX, the acidosis modulator has been reported in TNBC and RCC (Tan et al., 2009); further overexpression of epigenetic regulator (DNA methyltransferase, histone acetyltransferases, chromatin-remodelling enzymes, etc) and long-non coding RNA has been reported in gastric cancer, TNBC and PDAC (Krishnamachary et al., 2012; Onishi et al., 2012; Fujikuni et al., 2014; Liu et al., 2014; Wang et al., 2014); the chemokines are overexpressed in gastric cancer and multiple myeloma (Azab et al., 2012; Oh et al., 2012; Tao et al., 2014). Similarly, overexpression of cyclosporin binding protein cyclophilin A (CYPA) in PDAC (Zhang et al., 2014), endothelin in melanoma (Spinella et al., 2014); fascin in PDAC (Zhao et al., 2014); MMPs in PDAC, lung and ovarian cancer cell lines (Quintero-Fabián et al., 2019); protein kinase receptors in gastric, RCC, melanoma cancer (Chuang et al., 2008; Marconi et al., 2013) has been reported. HIF-1α also activates another critical cell signaling pathway i.e., HGF/MET signaling. Several studies suggest that MET, together with its ligand HGF, promotes cancer cell hallmarks including cell proliferation, survival, migration, angiogenesis in multiple mammalian cancer including hepatocellular carcinoma, head and neck cancer etc., (Goyal et al., 2013; Huang et al., 2020; Raj et al., 2022).

Additionally, in a positive feedback mechanism, ILK (Integrin Linked kinase) is activated by HIF-1α and is responsible for elevated HIF-1α expression through the regulatory loop (Matsuoka et al., 2013). Furthermore, E-cadherin, which was previously thought of as a tumor suppressor, was shown to have an unanticipated involvement in regulating genes involved in response to hypoxia and thus posing a potential role in metastatic breast cancer (Chu et al., 2013; Tam et al., 2020).

Moreover, intratumoral hypoxia alters the immune response of tumor in a variety of ways, all of which indicate an immunosuppressive impact (Palazón et al., 2012). HIF-1α, for example, can recruit myeloid-derived suppressor cells, regulatory T-cells, tumour-associated macrophages with immunosuppressive properties, as well as limit cytotoxic T-lymphocyte invasion (Corzo et al., 2010; Doedens et al., 2010; Imtiyaz et al., 2010; Barsoum et al., 2014). Besides that, HIF-1α stimulates the synthesis of the immunological checkpoint protein PD-L1(programmed death ligand-1), which aids in immune suppression and evasion (Noman et al., 2014; Abou Khouzam et al., 2021). As a result, the majority of the data implies that HIFs promote tumor growth through immunosuppression.

Collectively, these recent discoveries have motivated the scientific community to focus its efforts on developing novel drugs that can inhibit HIF-1α or its target genes. Further, we have focused on the compounds that have been developed as HIF-1α inhibitors and are now undergoing clinical trials. These novel compounds may pave the way for more effective therapy and might improve the prognosis of aggressive cancer patients.

### Advanced Clinical Trials Targeting the Adaption to Hypoxia Tumor Microenvironment Therapeutic Targets

The ability to specifically target cancer cells while causing minimal harm to normal cells is one of the “Holy Grail” of cancer therapy. The propensity to exploit abnormalities between normal and malignant cells has significantly aided the discovery of novel anti-cancer drugs. Various small compounds discovered have been briefly summarized in the following section, albeit the bulk of them are still in the early stages of clinical trials.

As discussed above, HIF-1α activation has been found to have a significant impact on cancer cell metabolism as it influences the expression of several genes leading to increased glycolysis and impaired mitochondrial function in tumor cells. Several anticancer drugs that modulate the activity or levels of HIF-1α in cells influence HIF-1 without directly targeting it.

Digoxin (DIG) (PubChem CID: 2724385), a cardiac glycoside, has been demonstrated to have an anti-cancer effect *in vitro* and *in vivo* in various solid tumors by inhibiting HIF-1α production (Newman et al., 2008; Zhang et al., 2008; Lin et al., 2009). DIG is now being studied in phase 2 clinical trial (https://clinicaltrials.gov/ct2/show/NCT01763931) as a new HIF-1α inhibitor in breast cancer. This clinical trial will also be valuable in evaluating adverse events, as well as the safety and tolerability of DIG in pre-surgical breast cancer patients using the Common Terminology Criteria for Adverse Events, version 4. Additionally, Ganetespib (PubChem CID: 135564985), (5-[2,4-dihyroxy-5-(1-methylethyl)phenyl]4-(1-methyl-1H-indol-5-yl)-2,4-dihydro-3H-1,2,4-triazol-3-one) have been reported to increase the proteasome-mediated degradation of Hsp90. Hsp90, a chaperone, is implicated in tumor development, angiogenesis, and the generation of cancer stem cells (Pillai and Ramalingam, 2014; White et al., 2016). Its route triggers the activation of multiple oncogenic proteins including HIF-1α. Thus by targeting Hsp90, Ganetespib inhibits HIF-1α in TNBC mouse model (Xiang et al., 2014). Ganetespib is now being studies in a phase 3 trial in patients with advanced non-small cell lung cancer (NSCLC) in conjunction with docetaxel (https://www.clinicaltrials.gov/ct2/show/NCT01798485). This clinical trial seeks to identify a potential synergism between ganetespib (150 mg/m^2^) and docetaxel (75 mg/m^2^) in order to suggest a more effective anti-cancer therapy than docetaxel alone.

Among multiple factors that influence hypoxia-induced tumor acidosis, CAIX is a hypoxia-inducible metal enzyme that promotes cancer cell survival/proliferation and invasion *via* HIF activation (Lock et al., 2013). It regulates cellular pH by catalyzing the reversible hydration of carbon dioxide to bicarbonate and protons. It is expressed exclusively on the cell surface of tumor cells, particularly CSCs (cancer stem cells), and is one of the key factors influencing cancer cell survival and metastasis (Lock et al., 2013). Moreover, CAIX is abundantly expressed in pancreatic ductal adenocarcinoma and breast cancer and has been implicated as a biomarker of poor prognosis for metastatic development and survival (Touisni et al., 2011; Lock et al., 2013). Additionally, research has proven a vital role for CAIX expression in the maintenance of the EMT phenotype, “stem cell” function, and hypoxia-induced tumor heterogeneity (Touisni et al., 2011; Ledaki et al., 2015). SLC-0111 (PubChem CID: 310360) is a small molecule that reaches the hypoxic niches and selectively binds and inhibits CAIX. Presently SLC-0111 is in phase I clinical trial (https://clinicaltrials.gov/ct2/show/NCT02215850) and the study focuses on its safety, tolerability, and pharmacokinetics, and efficacy in treating cancers. Similarly, another molecule DTP348 (PubChem CID: 57413968) namely 2-(2-methyl-5-nitro-1H-imidazol-1-yl) ethylsulfamide, is reported to target CIAX (Rami et al., 2013). Presently, this oral dual CAIX inhibitor/radiosensitizer is being researched in phase I clinical trial (https://clinicaltrials.gov/ct2/show/NCT02216669). This clinical study will consider the effects of DTP348 alone and in combination with radiation in patients with solid tumors to establish the appropriate phase II clinical trial dosage, safety, and tolerability.

Interestingly, HGF is the natural ligand of *MET,* a proto-oncogene. The HIF-1α induced HGF/MET pathway activation has been reported to induce EMT transition, resulting in a mesenchymal population that is more tumorigenic and chemoresistant than the preceding ones (Cañadas et al., 2014). Rilotumumab, Crizotinib/axitinib and cabozantinib are designed to effectively target HGF/MET pathway. Rilotumumab (PubChem SID: 135262715), is a human monoclonal antibody that is reported to significantly block the binding of HGF/SF to its MET receptor. Presently, it is being tested in phase 3 clinical trial (https://clinicaltrials.gov/ct2/show/NCT01697072) to evaluate if the treatment with epirubicin, cisplatin, and capecitabine in combination with rilotumumab results in better clinical outcomes in metastatic *MET* positive gastric cancers. Axitinib (PubChem CID: 6450551), with crizotinib (PubChem CID: 11626560), is currently being tested in a phase 1b clinical trial in patients with advanced solid tumors (https://clinicaltrials.gov/ct2/show/NCT01999972) (Kwak et al., 2010; Chen Y. et al., 2015). Moreover, cabozantinib is an oral inhibitor of *MET*, *RET, ROS1, NTRK1,* and AXL. It has been found to shrink tumor cells and significantly reduce cellular proliferation in medullary thyroid and prostate cancer. Cabozantinib (PubChem CID: 46830297), is currently being investigated to determine objective response rate (ORR), overall survival (OS) and progression-free survival (PFS) in advanced non-small cell lung cancer with *RET* fusions and those with *ROS1* or *NTRK1* fusions or elevated *MET* or *AXL* activity (https://clinicaltrials.gov/ct2/show/NCT01639508).

According to the current research, several phytocompounds also have been shown to play a significant role in cancer therapy and have numerous potential targets in tumorigenesis, including HIF-1 (Deng et al., 2019). Baicalein (PubChem CID: 5281605), (5,6,7- trihydroxyflavone), a flavonoid derived from *Scutellaria baicalensis* has been reported to have potent cytotoxic activity against a wide range of cancer (Bie et al., 2017; Dou et al., 2018; Wang et al., 2019). Surprisingly, baicalein when administered leads to the inhibition of hypoxia-induced Akt phosphorylation as a result of increased PTEN accumulation and decreased HIF-1α expression. Thus baicalein is a potential therapeutic sensitiser against gastric cancer since it inhibits glycolysis *via* PTEN/Akt/HIF-1α (Chen F. et al., 2015). Other investigations have corroborated the inhibitory effects of phytochemicals on HIF-1 in control of glucose metabolism. For instance, methylalpinumisoflavone (MF) (PubChem CID: 15596285), a flavonoid isolated from *Lanchocarpus glabrescens,* demonstrates a strong anti-cancer effect on T47D cells by suppressing HIF-1 and targets genes including CDKN1A, VEGF, and GLUT-1 in T47D cells (Liu et al., 2009). Moreover, oroxylin A (PubChem CID: 5320315) treatment has been linked to a reduction in cancer-related glycolysis *via* sirtuin-3 mediated destabilization of HIF-1 in MDA-MB-231 cells (Wei et al., 2015). Furthermore, EGCG (PubChem CID: 65064) is known to decrease the HIF-1α and glycolysis-related enzymes in T47D cells (Wei et al., 2018). Additionally, resveratrol (PubChem CID: 445154) has been shown to reduce the cellular uptake of glucose and induce glycolysis in cancer cell lines. Resveratrol inhibited intracellular reactive oxidative species (ROS) and hence lowered HIF-1 accumulation, decreased GLUT-1 expression, and induced glycolytic flow, according to measurements of cellular absorption of the glucose analogue 18F-fluorodeoxyglucose following resveratrol exposure (Jung et al., 2013).

Further using a combination of anti-cancer therapies is more likely to be successful than using a single drug (Maschek et al., 2004). Another concept has been proposed that takes the use of underlying metabolic variations between malignancies and healthy tissues (Payne, 2007). For instance, many tumors’ reliance on glycolysis has been addressed using a variety of glycolytic pathway enzyme inhibitors that are also being evaluated as possible treatment drugs (Maher et al., 2004; Maschek et al., 2004; Xu et al., 2005; Pelicano et al., 2006; Gogvadze et al., 2009; Marín-Hernández et al., 2009; Mathupala et al., 2009). The major targets thus far have been glucose absorption (mediated mostly by GLUT-1), glucose retention (mediated by hexokinase) and lactate generation (catalyzed by lactate dehydrogenase-A). Unfortunately, inhibiting glycolysis has a significant complication; unlike organs that may easily utilise carbon sources other than glucose, the brain, retina, and testes are extremely glucose dependent. As a result, different metabolic targets such as specific glycolytic pathway enzyme isoforms which are transcriptionally overexpressed in response to HIF-1 elevations must be taken into account (Marín-Hernández et al., 2009). Targeting proteins such as GLUTs, HK1, HKII, PFK-L, ALD-A, ALD-C, PGK1, ENO-α, PYK-M2, LDH-A, PFKFB-3 along with HIF-1α may be more trackable for drug development than HIF-1α itself. Identifying metabolic alterations that are specific to malignancies is inevitably a critical research goal.

## Conclusion

Metabolic reprogramming is a frequent cancer cell mechanism for dealing with elevated energy demands. The growing interest in cancer metabolism has already resulted in a slew of novel potential therapeutics. In conclusion, several reports have shown that hypoxic cells may adapt to low oxygen levels by changing transcriptional and translational responses to increase glucose absorption and anaerobic catabolism. Since HIF-1 has been proven to be a master regulator of a wide range of proteins and enzymes involved in glucose metabolism and the glycolytic pathway. Thus modulation of the HIF-1 pathway is a promising therapeutic strategy. It is envisaged that a deeper insight into the molecular mechanisms involved in HIF-1 regulation and the Warburg effect in carcinogenesis would unlock new therapeutic interventions. Nonetheless, due to the present generation of agents’ limited selectivity and specificity, there are possible challenges and concerns. Additionally, the recent metabolism-based therapeutics have shown some harmful effects on normal cells. Therefore, we propose combining the drugs to target distinct elements of cancer bioenergetics and hypoxia-induced factors in order to develop synergistic cancer treatments. Furthermore, directing these molecules to their targets would limit off-target effects while increasing efficacy.

## Glossary

ATP: adenosine triphosphate
AXL: AXL receptor tyrosine kinase
BECN1: beclin-1
CAIX: carbonic anhydrase 9 precursor
CRC: colorectal cancer
DIG: digoxin
EGCG: epigallocatechin gallate
EMT: epithelial-mesenchymal transition
ENO-α: alpha-enolase
GLUT: glucose transporter
HIF: 1α-hypoxia-inducible factor-1α
HIF-1: hypoxia-inducible factor-1
HGF: hepatocyte growth factor
HK1: hexokinase-1
hsp90: heat shock protein 90
HTM: hypoxic tumor microenvironment
ILK: Integrin Linked kinase
LDH: lactate dehydrogenase
MAP1LC3: microtubule-associated proteins 1A/1B light chain 3B
MF: methylalpinumisoflavone
MET: mesenchymal-epithelial transition
NTRK1: neurotrophic receptor tyrosine kinase 1
NSCLC: non-small lung cancer
OXPHOS: oxidative phosphorylation
PDAC: pancreatic ductal adenocarcinoma
PD-L1: programmed death ligand-1
RCC: renal cell carcinoma
RNA: ribonucleic acid
RET: rearranged during transfection
PGK1: phosphoglycerate kinase 1
PFKFB: 6-phosphofructo-2-kinase/fructose-2,6-biphosphatase 3
PFK: Phosphofructokinase
PTEN: phosphatase and tensin homolog
PYK: M2-M2 isoform of pyruvate kinase
ROS1: ROS proto-oncogene 1
TME: tumor microenvironment
TNBC: triple-negative breast cancer
VEGF: vascular endothelial growth factor
VHL: Von Hippel-Lindau

## References

[B1] Abou Khouzam R. Brodaczewska K. Filipiak A. Zeinelabdin N. A. Buart S. Szczylik C. (2021). Tumor Hypoxia Regulates Immune Escape/Invasion: Influence on Angiogenesis and Potential Impact of Hypoxic Biomarkers on Cancer Therapies. Front. Immunol. 11. 10.3389/fimmu.2020.613114 PMC7854546 33552076

[B2] Arora A. Singh S. Bhatt A. N. Pandey S. Sandhir R. Dwarakanath B. S. (2015). Interplay between Metabolism and Oncogenic Process: Role of microRNAs. Translational oncogenomics 7, 11–27. 10.4137/TOG.S29652 26740741 PMC4696840

[B3] Azab A. K. Hu J. Quang P. Azab F. Pitsillides C. Awwad R. (2012). Hypoxia Promotes Dissemination of Multiple Myeloma through Acquisition of Epithelial to Mesenchymal Transition-like Features. Blood 119, 5782–5794. 10.1182/BLOOD-2011-09-380410 22394600 PMC3382938

[B4] Barsoum I. B. Smallwood C. A. Siemens D. R. Graham C. H. (2014). A Mechanism of Hypoxia-Mediated Escape from Adaptive Immunity in Cancer Cells. Cancer Res. 74, 665–674. 10.1158/0008-5472.CAN-13-0992 24336068

[B5] Bie B. Sun J. Guo Y. Li J. Jiang W. Yang J. (2017). Baicalein: A Review of its Anti-cancer Effects and Mechanisms in Hepatocellular Carcinoma. Biomed. Pharmacother. 93, 1285–1291. 10.1016/j.biopha.2017.07.068 28747003

[B6] Bos R. Zhong H. Hanrahan C. F. Mommers E. C. M. Semenza G. L. Pinedo H. M. (2001). Levels of Hypoxia-Inducible Factor-1 during Breast Carcinogenesis. JNCI J. Natl. Cancer Inst. 93, 309–314. 10.1093/jnci/93.4.309 11181778

[B7] Cañadas I. Rojo F. Taus Á. Arpí O. Arumí-Uría M. Pijuan L. (2014). Targeting Epithelial-To-Mesenchymal Transition with Met Inhibitors Reverts Chemoresistance in Small Cell Lung Cancer. Clin. Cancer Res. 20, 938–950. 10.1158/1078-0432.CCR-13-1330 24284055

[B8] Carmeliet P. Dor Y. Herbert J.-M. Fukumura D. Brusselmans K. Dewerchin M. (1998). Role of HIF-1α in Hypoxia-Mediated Apoptosis, Cell Proliferation and Tumour Angiogenesis. Nature 394, 485–490. 10.1038/28867 9697772

[B9] Casero R. A. Pegg A. E. (2009). Polyamine Catabolism and Disease. Biochem. J. 421, 323–338. 10.1042/BJ20090598 19589128 PMC2756025

[B10] Chaffer C. L. Weinberg R. A. (2011). A Perspective on Cancer Cell Metastasis. Science 331, 1559–1564. 10.1126/SCIENCE.1203543 21436443

[B11] Chen F. Zhuang M. Zhong C. Peng J. Wang X. Li J. (2015a). Baicalein Reverses Hypoxia-Induced 5-FU Resistance in Gastric Cancer AGS Cells through Suppression of Glycolysis and the PTEN/Akt/HIF-1α Signaling Pathway. Oncol. Rep. 33, 457–463. 10.3892/or.2014.3550 25333894

[B12] Chen H. H. W. Su W.-C. Lin P.-W. Guo H.-R. Lee W.-Y. (2007). Hypoxia-inducible Factor-1α Correlates with MET and Metastasis in Node-Negative Breast Cancer. Breast Cancer Res. Treat. 103, 167–175. 10.1007/s10549-006-9360-3 17028975

[B13] Chen Y. Suzuki A. Tortorici M. A. Garrett M. LaBadie R. R. Umeyama Y. (2015b). Axitinib Plasma Pharmacokinetics and Ethnic Differences. Invest. New Drugs 33, 521–532. 10.1007/s10637-015-0214-x 25663295

[B14] Chou C.-W. Wang C.-C. Wu C.-P. Lin Y.-J. Lee Y.-C. Cheng Y.-W. (2012). Tumor Cycling Hypoxia Induces Chemoresistance in Glioblastoma Multiforme by Upregulating the Expression and Function of ABCB1. Neuro-Oncology 14, 1227–1238. 10.1093/NEUONC/NOS195 22946104 PMC3452342

[B15] Chu K. Boley K. M. Moraes R. Barsky S. H. Robertson F. M. (2013). The Paradox of E-Cadherin: Role in Response to Hypoxia in the Tumor Microenvironment and Regulation of Energy Metabolism. Oncotarget 4, 446–462. 10.18632/ONCOTARGET.872 23530113 PMC3717307

[B16] Chuang M.-J. Sun K.-H. Tang S.-J. Deng M.-W. Wu Y.-H. Sung J.-S. (2008). Tumor-derived Tumor Necrosis Factor-Alpha Promotes Progression and Epithelial-Mesenchymal Transition in Renal Cell Carcinoma Cells. Cancer Sci. 99, 905–913. 10.1111/J.1349-7006.2008.00756.X 18294286 PMC11158824

[B17] Corzo C. A. Condamine T. Lu L. Cotter M. J. Youn J.-I. Cheng P. (2010). HIF-1α Regulates Function and Differentiation of Myeloid-Derived Suppressor Cells in the Tumor Microenvironment. J. Exp. Med. 207, 2439–2453. 10.1084/jem.20100587 20876310 PMC2964584

[B18] Dales J.-P. Garcia S. Meunier-Carpentier S. Andrac-Meyer L. Haddad O. Lavaut M.-N. (2005). Overexpression of Hypoxia-Inducible Factor HIF-1α Predicts Early Relapse in Breast Cancer: Retrospective Study in a Series of 745 Patients. Int. J. Cancer 116, 734–739. 10.1002/ijc.20984 15849727

[B19] Deng X. Peng Y. Zhao J. Lei X. Zheng X. Xie Z. (2020). Anticancer Activity of Natural Flavonoids: Inhibition of HIF-1α Signaling Pathway. Coc 23, 2945–2959. 10.2174/1385272823666191203122030

[B20] Denko N. C. (2008). Hypoxia, HIF1 and Glucose Metabolism in the Solid Tumour. Nat. Rev. Cancer 8, 705–713. 10.1038/nrc2468 19143055

[B21] Doedens A. L. Stockmann C. Rubinstein M. P. Liao D. Zhang N. DeNardo D. G. (2010). Macrophage Expression of Hypoxia-Inducible Factor-1α Suppresses T-Cell Function and Promotes Tumor Progression. Cancer Res. 70, 7465–7475. 10.1158/0008-5472.CAN-10-1439 20841473 PMC2948598

[B22] Dou J. Wang Z. Ma L. Peng B. Mao K. Li C. (2018). Baicalein and Baicalin Inhibit colon Cancer Using Two Distinct Fashions of Apoptosis and Senescence. Oncotarget 9, 20089–20102. 10.18632/oncotarget.24015 29732005 PMC5929448

[B23] Frezza C. Gottlieb E. (2009). Mitochondria in Cancer: Not Just Innocent Bystanders. Semin. Cancer Biol. 19, 4–11. 10.1016/j.semcancer.2008.11.008 19101633

[B24] Fujikuni N. Yamamoto H. Tanabe K. Naito Y. Sakamoto N. Tanaka Y. (2014). Hypoxia-mediated CD24 Expression Is Correlated with Gastric Cancer Aggressiveness by Promoting Cell Migration and Invasion. Cancer Sci. 105, 1411–1420. 10.1111/CAS.12522 25174257 PMC4462374

[B25] Gatenby R. A. Gillies R. J. (2004). Why Do Cancers Have High Aerobic Glycolysis? Nat. Rev. Cancer 4, 891–899. 10.1038/nrc1478 15516961

[B26] Gatenby R. A. Smallbone K. Maini P. K. Rose F. Averill J. Nagle R. B. (2007). Cellular Adaptations to Hypoxia and Acidosis during Somatic Evolution of Breast Cancer. Br. J. Cancer 97, 646–653. 10.1038/sj.bjc.6603922 17687336 PMC2360372

[B27] Gogvadze V. Orrenius S. Zhivotovsky B. (2009). Mitochondria as Targets for Cancer Chemotherapy. Semin. Cancer Biol. 19, 57–66. 10.1016/j.semcancer.2008.11.007 19101636

[B28] Goyal L. Muzumdar M. D. Zhu A. X. (2013). Targeting the HGF/c-MET Pathway in Hepatocellular Carcinoma. Clin. Cancer Res. 19, 2310–2318. 10.1158/1078-0432.CCR-12-2791 23388504 PMC4583193

[B29] Hanahan D. (2022). Hallmarks of Cancer: New Dimensions. Cancer Discov. 12, 31–46. 10.1158/2159-8290.CD-21-1059 35022204

[B30] Hanahan D. Weinberg R. A. (2011). Hallmarks of Cancer: The Next Generation. Cell 144, 646–674. 10.1016/j.cell.2011.02.013 21376230

[B31] Heldin C.-H. Rubin K. Pietras K. Östman A. (2004). High Interstitial Fluid Pressure - an Obstacle in Cancer Therapy. Nat. Rev. Cancer 4, 806–813. 10.1038/nrc1456 15510161

[B32] Huang X. Li E. Shen H. Wang X. Tang T. Zhang X. (2020). Targeting the HGF/MET Axis in Cancer Therapy: Challenges in Resistance and Opportunities for Improvement. Front. Cel Dev. Biol. 8. 10.3389/fcell.2020.00152 PMC7218174 32435640

[B33] Imtiyaz H. Z. Williams E. P. Hickey M. M. Patel S. A. Durham A. C. Yuan L.-J. (2010). Hypoxia-inducible Factor 2α Regulates Macrophage Function in Mouse Models of Acute and Tumor Inflammation. J. Clin. Invest. 120, 2699–2714. 10.1172/JCI39506 20644254 PMC2912179

[B34] Jeon S.-M. Hay N. (2018). Expanding the Concepts of Cancer Metabolism. Exp. Mol. Med. 50, 1–3. 10.1038/s12276-018-0070-9 PMC5938029 29657329

[B35] Jung K.-H. Lee J. H. Thien Quach C. H. Paik J.-Y. Oh H. Park J. W. (2013). Resveratrol Suppresses Cancer Cell Glucose Uptake by Targeting Reactive Oxygen Species-Mediated Hypoxia-Inducible Factor-1α Activation. J. Nucl. Med. 54, 2161–2167. 10.2967/jnumed.112.115436 24221993

[B36] Kalluri R. Weinberg R. A. (2009). The Basics of Epithelial-Mesenchymal Transition. J. Clin. Invest. 119, 1420–1428. 10.1172/JCI39104 19487818 PMC2689101

[B37] Ke Q. Costa M. (2006). Hypoxia-inducible Factor-1 (HIF-1). Mol. Pharmacol. 70, 1469–1480. 10.1124/mol.106.027029 16887934

[B38] Kogita A. Togashi Y. Hayashi H. Sogabe S. Terashima M. de Velasco M. A. (2014). Hypoxia Induces Resistance to ALK Inhibitors in the H3122 Non-small Cell Lung Cancer Cell Line with an ALK Rearrangement *via* Epithelial-Mesenchymal Transition. Int. J. Oncol. 45, 1430–1436. 10.3892/IJO.2014.2574 25096400 PMC4151805

[B39] Krishnamachary B. Penet M.-F. Nimmagadda S. Mironchik Y. Raman V. Solaiyappan M. (2012). Hypoxia Regulates CD44 and its Variant Isoforms through HIF-1α in Triple Negative Breast Cancer. PloS one 7, e44078. 10.1371/JOURNAL.PONE.0044078 22937154 PMC3429433

[B40] Kwak E. L. Bang Y.-J. Camidge D. R. Shaw A. T. Solomon B. Maki R. G. (2010). Anaplastic Lymphoma Kinase Inhibition in Non-small-cell Lung Cancer. N. Engl. J. Med. 363, 1693–1703. 10.1056/NEJMoa1006448 20979469 PMC3014291

[B41] Ledaki I. McIntyre A. Wigfield S. Buffa F. McGowan S. Baban D. (2015). Carbonic Anhydrase IX Induction Defines a Heterogeneous Cancer Cell Response to Hypoxia and Mediates Stem Cell-like Properties and Sensitivity to HDAC Inhibition. Oncotarget 6, 19413–19427. 10.18632/oncotarget.4989 26305601 PMC4637295

[B42] Lin J. Denmeade S. Carducci M. (2009). HIF-1α and Calcium Signaling as Targets for Treatment of Prostate Cancer by Cardiac Glycosides. Ccdt 9, 881–887. 10.2174/156800909789760249 20025575

[B43] Liu N. Wang Y. Zhou Y. Pang H. Zhou J. Qian P. (2014). Krüppel-like Factor 8 Involved in Hypoxia Promotes the Invasion and Metastasis of Gastric Cancer *via* Epithelial to Mesenchymal Transition. Oncol. Rep. 32, 2397–2404. 10.3892/OR.2014.3495 25333643

[B44] Liu S. Kumar S. M. Martin J. S. Yang R. Xu X. (2011). Snail1 Mediates Hypoxia-Induced Melanoma Progression. Am. J. Pathol. 179, 3020–3031. 10.1016/J.AJPATH.2011.08.038 21996677 PMC3260799

[B45] Liu Y. Veena C. K. Morgan J. B. Mohammed K. A. Jekabsons M. B. Nagle D. G. (2009). Methylalpinumisoflavone Inhibits Hypoxia-Inducible Factor-1 (HIF-1) Activation by Simultaneously Targeting Multiple Pathways. J. Biol. Chem. 284, 5859–5868. 10.1074/jbc.M806744200 19091749 PMC2645834

[B46] Lock F. E. McDonald P. C. Lou Y. Serrano I. Chafe S. C. Ostlund C. (2013). Targeting Carbonic Anhydrase IX Depletes Breast Cancer Stem Cells within the Hypoxic Niche. Oncogene 32, 5210–5219. 10.1038/onc.2012.550 23208505

[B47] Maher J. C. Krishan A. Lampidis T. J. (2004). Greater Cell Cycle Inhibition and Cytotoxicity Induced by 2-Deoxy-D-Glucose in Tumor Cells Treated under Hypoxic vs Aerobic Conditions. Cancer Chemother. Pharmacol. 53, 116–122. 10.1007/s00280-003-0724-7 14605866

[B48] Marconi C. Peppicelli S. Bianchini F. Calorini L. (2013). TNFα Receptor1 Drives Hypoxia-Promoted Invasiveness of Human Melanoma Cells. Exp. Oncol. 35, 187–191. Available at: https://pubmed.ncbi.nlm.nih.gov/24084456/(Accessed December 28, 2021). 24084456

[B49] Marin-Hernandez A. Gallardo-Perez J. Ralph S. Rodriguez-Enriquez S. Moreno-Sanchez R. (2009). HIF-1α Modulates Energy Metabolism in Cancer Cells by Inducing Over-expression of Specific Glycolytic Isoforms. Mrmc 9, 1084–1101. 10.2174/138955709788922610 19689405

[B50] Maschek G. Savaraj N. Priebe W. Braunschweiger P. Hamilton K. Tidmarsh G. F. (2004). 2-deoxy-D-glucose Increases the Efficacy of Adriamycin and Paclitaxel in Human Osteosarcoma and Non-small Cell Lung Cancers *In Vivo* . Cancer Res. 64, 31–34. 10.1158/0008-5472.can-03-3294 14729604

[B51] Mathupala S. P. Ko Y. H. Pedersen P. L. (2009). Hexokinase-2 Bound to Mitochondria: Cancer's Stygian Link to the "Warburg Effect" and a Pivotal Target for Effective Therapy. Semin. Cancer Biol. 19, 17–24. 10.1016/j.semcancer.2008.11.006 19101634 PMC2714668

[B52] Matsuoka J. Yashiro M. Doi Y. Fuyuhiro Y. Kato Y. Shinto O. (2013). Hypoxia Stimulates the EMT of Gastric Cancer Cells through Autocrine TGFβ Signaling. PLoS ONE 8, e62310. 10.1371/JOURNAL.PONE.0062310 23690936 PMC3656884

[B53] Newman R. A. Yang P. Pawlus A. D. Block K. I. (2008). Cardiac Glycosides as Novel Cancer Therapeutic Agents. Mol. interventions 8, 36–49. 10.1124/mi.8.1.8 18332483

[B54] Noman M. Z. Desantis G. Janji B. Hasmim M. Karray S. Dessen P. (2014). PD-L1 Is a Novel Direct Target of HIF-1α, and its Blockade under Hypoxia Enhanced MDSC-Mediated T Cell Activation. J. Exp. Med. 211, 781–790. 10.1084/jem.20131916 24778419 PMC4010891

[B55] Oh Y. S. Kim H. Y. Song I.-C. Yun H.-J. Jo D.-Y. Kim S. (2012). Hypoxia Induces CXCR4 Expression and Biological Activity in Gastric Cancer Cells through Activation of Hypoxia-Inducible Factor-1α. Oncol. Rep. 28, 2239–2246. 10.3892/OR.2012.2063 23023480

[B56] Onishi H. Morifuji Y. Kai M. Suyama K. Iwasaki H. Katano M. (2012). Hedgehog Inhibitor Decreases Chemosensitivity to 5-fluorouracil and Gemcitabine under Hypoxic Conditions in Pancreatic Cancer. Cancer Sci. 103, 1272–1279. 10.1111/J.1349-7006.2012.02297.X 22486854 PMC7659296

[B57] Otto A. M. (2016). Warburg Effect(s)-A Biographical Sketch of Otto Warburg and His Impacts on Tumor Metabolism. Cancer Metab. 4, 5. 10.1186/s40170-016-0145-9 26962452 PMC4784299

[B58] Palazón A. Aragonés J. Morales-Kastresana A. de Landázuri M. O. Melero I. (2012). Molecular Pathways: Hypoxia Response in Immune Cells Fighting or Promoting Cancer. Clin. Cancer Res. 18, 1207–1213. 10.1158/1078-0432.CCR-11-1591 22205687

[B59] Payne A. G. (2007). Exploiting Hypoxia in Solid Tumors to Achieve Oncolysis. Med. hypotheses 68, 828–831. 10.1016/j.mehy.2006.09.013 17055180

[B60] Pelicano H. Martin D. S. Xu R.-H. Huang P. (2006). Glycolysis Inhibition for Anticancer Treatment. Oncogene 25, 4633–4646. 10.1038/sj.onc.1209597 16892078

[B61] Pillai R. N. Ramalingam S. S. (2014). Heat Shock Protein 90 Inhibitors in Non-small-cell Lung Cancer. Curr. Opin. Oncol. 26, 159–164. 10.1097/CCO.0000000000000047 24463348

[B62] Platten M. Wick W. van den Eynde B. J. (2012). Tryptophan Catabolism in Cancer: beyond Ido and Tryptophan Depletion. Cancer Res. 72, 5435–5440. 10.1158/0008-5472.CAN-12-0569 23090118

[B63] Quintero-Fabián S. Arreola R. Becerril-Villanueva E. Torres-Romero J. C. Arana-Argáez V. Lara-Riegos J. (2019). Role of Matrix Metalloproteinases in Angiogenesis and Cancer. Front. Oncol. 9, 1370. 10.3389/FONC.2019.01370/BIBTEX 31921634 PMC6915110

[B64] Raj S. Kesari K. K. Kumar A. Rathi B. Sharma A. Gupta P. K. (2022). Molecular Mechanism(s) of Regulation(s) of C-MET/HGF Signaling in Head and Neck Cancer. Mol. Cancer 21, 31. 10.1186/s12943-022-01503-1 35081970 PMC8790852

[B65] Rami M. Dubois L. Parvathaneni N.-K. Alterio V. van Kuijk S. J. A. Monti S. M. (2013). Hypoxia-targeting Carbonic Anhydrase IX Inhibitors by a New Series of Nitroimidazole-Sulfonamides/sulfamides/sulfamates. J. Med. Chem. 56, 8512–8520. 10.1021/jm4009532 24128000

[B66] Semenza G. L. Roth P. H. Fang H. M. Wang G. L. (1994). Transcriptional Regulation of Genes Encoding Glycolytic Enzymes by Hypoxia-Inducible Factor 1. J. Biol. Chem. 269, 23757–23763. 10.1016/s0021-9258(17)31580-6 8089148

[B67] Simiantonaki N. Taxeidis M. Jayasinghe C. Kurzik-Dumke U. Kirkpatrick C. J. (2008). Hypoxia-inducible Factor 1 Alpha Expression Increases during Colorectal Carcinogenesis and Tumor Progression. BMC Cancer 8, 320. 10.1186/1471-2407-8-320 18983642 PMC2584660

[B68] Singh A. Settleman J. (2010). EMT, Cancer Stem Cells and Drug Resistance: an Emerging axis of Evil in the War on Cancer. Oncogene 29, 4741–4751. 10.1038/ONC.2010.215 20531305 PMC3176718

[B69] Sørensen B. S. Horsman M. R. (2020). Tumor Hypoxia: Impact on Radiation Therapy and Molecular Pathways. Front. Oncol. 10. 10.3389/fonc.2020.00562 PMC7186437 32373534

[B70] Spinella F. Caprara V. Cianfrocca R. Rosanò L. di Castro V. Garrafa E. (2014). The Interplay between Hypoxia, Endothelial and Melanoma Cells Regulates Vascularization and Cell Motility through Endothelin-1 and Vascular Endothelial Growth Factor. Carcinogenesis 35, 840–848. 10.1093/CARCIN/BGU018 24473118 PMC3988429

[B71] Sutherland R. M. (1988). Cell and Environment Interactions in Tumor Microregions: the Multicell Spheroid Model. Science 240, 177–184. 10.1126/SCIENCE.2451290 2451290

[B72] Tam S. Y. Wu V. W. C. Law H. K. W. (2020). Hypoxia-Induced Epithelial-Mesenchymal Transition in Cancers: HIF-1α and beyond. Front. Oncol. 10. 10.3389/fonc.2020.00486 PMC7156534 32322559

[B73] Tan E. Y. Yan M. Campo L. Han C. Takano E. Turley H. (2009). The Key Hypoxia Regulated Gene CAIX Is Upregulated in Basal-like Breast Tumours and Is Associated with Resistance to Chemotherapy. Br. J. Cancer 100, 405–411. 10.1038/SJ.BJC.6604844 19165203 PMC2634728

[B74] Tao L.-L. Shi S. J. Chen L. B. Huang G. C. (2014). Expression of Monocyte Chemotactic protein-1/CCL2 in Gastric Cancer and its Relationship with Tumor Hypoxia. Wjg 20, 4421–4427. 10.3748/WJG.V20.I15.4421 24764682 PMC3989980

[B75] Touisni N. Maresca A. McDonald P. C. Lou Y. Scozzafava A. Dedhar S. (2011). Glycosyl Coumarin Carbonic Anhydrase IX and XII Inhibitors Strongly Attenuate the Growth of Primary Breast Tumors. J. Med. Chem. 54, 8271–8277. 10.1021/jm200983e 22077347

[B76] Valastyan S. Weinberg R. A. (2011). Tumor Metastasis: Molecular Insights and Evolving Paradigms. Cell 147, 275–292. 10.1016/J.CELL.2011.09.024 22000009 PMC3261217

[B77] Vaupel P. Multhoff G. (2021). Revisiting the Warburg Effect: Historical Dogma versus Current Understanding. J. Physiol. 599, 1745–1757. 10.1113/JP278810 33347611

[B78] Vaupel P. (2004). The Role of Hypoxia-Induced Factors in Tumor Progression. The oncologist 9 (Suppl. 5), 10–17. 10.1634/THEONCOLOGIST.9-90005-10 15591418

[B79] Wang G. L. Jiang B. H. Rue E. A. Semenza G. L. (1995). Hypoxia-inducible Factor 1 Is a basic-helix-loop-helix-PAS Heterodimer Regulated by Cellular O2 Tension. Proc. Natl. Acad. Sci. 92, 5510–5514. 10.1073/pnas.92.12.5510 7539918 PMC41725

[B80] Wang M. Qiu S. Qin J. (2019). Baicalein Induced Apoptosis and Autophagy of Undifferentiated Thyroid Cancer Cells by the ERK/PI3K/Akt Pathway. Am. J. Transl Res. 11, 3341–3352. 31312348 PMC6614652

[B81] Wang Y. Liu X. Zhang H. Sun L. Zhou Y. Jin H. (2014). Hypoxia-Inducible lncRNA-AK058003 Promotes Gastric Cancer Metastasis by Targeting γ-Synuclein. Neoplasia 16, 1094–1106. 10.1016/J.NEO.2014.10.008 25499222 PMC4309257

[B82] Wei L. Zhou Y. Qiao C. Ni T. Li Z. You Q. (2015). Oroxylin A Inhibits Glycolysis-dependent Proliferation of Human Breast Cancer *via* Promoting SIRT3-Mediated SOD2 Transcription and HIF1α Destabilization. Cell Death Dis 6–e1714. 10.1038/cddis.2015.86 PMC4650553 25855962

[B83] Wei R. Mao L. Xu P. Zheng X. Hackman R. M. Mackenzie G. G. (2018). Suppressing Glucose Metabolism with Epigallocatechin-3-Gallate (EGCG) Reduces Breast Cancer Cell Growth in Preclinical Models. Food Funct. 9, 5682–5696. 10.1039/c8fo01397g 30310905 PMC7480214

[B84] White P. T. Subramanian C. Zhu Q. Zhang H. Zhao H. Gallagher R. (2016). Novel HSP90 Inhibitors Effectively Target Functions of Thyroid Cancer Stem Cell Preventing Migration and Invasion. Surgery 159, 142–151. 10.1016/j.surg.2015.07.050 26542767 PMC4709031

[B85] Wigerup C. Påhlman S. Bexell D. (2016). Therapeutic Targeting of Hypoxia and Hypoxia-Inducible Factors in Cancer. Pharmacol. Ther. 164, 152–169. 10.1016/j.pharmthera.2016.04.009 27139518

[B86] Xiang L. Gilkes D. M. Chaturvedi P. Luo W. Hu H. Takano N. (2014). Ganetespib Blocks HIF-1 Activity and Inhibits Tumor Growth, Vascularization, Stem Cell Maintenance, Invasion, and Metastasis in Orthotopic Mouse Models of Triple-Negative Breast Cancer. J. Mol. Med. 92, 151–164. 10.1007/s00109-013-1102-5 24248265 PMC3946681

[B87] Xu R. H. Pelicano H. Zhou Y. Carew J. S. Feng L. Bhalla K. N. (2005). Inhibition of Glycolysis in Cancer Cells: a Novel Strategy to Overcome Drug Resistance Associated with Mitochondrial Respiratory Defect and Hypoxia. Cancer Res. 65, 613–621. 10.1158/0008-5472.613.65.2 15695406

[B88] Yang M.-H. Wu M.-Z. Chiou S.-H. Chen P.-M. Chang S.-Y. Liu C.-J. (2008). Direct Regulation of TWIST by HIF-1α Promotes Metastasis. Nat. Cel Biol 10, 295–305. 10.1038/NCB1691 18297062

[B89] Yang Y. J. Na H. J. Suh M. J. Ban M. J. Byeon H. K. Kim W. S. (2015). Hypoxia Induces Epithelial-Mesenchymal Transition in Follicular Thyroid Cancer: Involvement of Regulation of Twist by Hypoxia Inducible Factor-1α. Yonsei Med. J. 56, 1503. 10.3349/YMJ.2015.56.6.1503 26446630 PMC4630036

[B90] Zhang F. Du G. (2012). Dysregulated Lipid Metabolism in Cancer. Wjbc 3, 167–174. 10.4331/wjbc.v3.i8.167 22937213 PMC3430731

[B91] Zhang H. Chen J. Liu F. Gao C. Wang X. Zhao T. (2014). CypA, a Gene Downstream of HIF-1α, Promotes the Development of PDAC. PloS one 9, e92824. 10.1371/JOURNAL.PONE.0092824 24662981 PMC3963943

[B92] Zhang H. Qian D. Z. Tan Y. S. Lee K. Gao P. Ren Y. R. (2008). Digoxin and Other Cardiac Glycosides Inhibit HIF-1α Synthesis and Block Tumor Growth. Proc. Natl. Acad. Sci. U.S.A. 105, 19579–19586. 10.1073/pnas.0809763105 19020076 PMC2604945

[B93] Zhao X. Gao S. Ren H. Sun W. Zhang H. Sun J. (2014). Hypoxia-inducible Factor-1 Promotes Pancreatic Ductal Adenocarcinoma Invasion and Metastasis by Activating Transcription of the Actin-Bundling Protein Fascin. Cancer Res. 74, 2455–2464. 10.1158/0008-5472.CAN-13-3009 24599125

[B94] Zhou J. Schmid T. Schnitzer S. Brüne B. (2006). Tumor Hypoxia and Cancer Progression. Cancer Lett. 237, 10–21. 10.1016/j.canlet.2005.05.028 16002209

[B95] Zhu H. Wang D. Zhang L. Xie X. Wu Y. Liu Y. (2014). Upregulation of Autophagy by Hypoxia-Inducible Factor-1α Promotes EMT and Metastatic Ability of CD133+ Pancreatic Cancer Stem-like Cells during Intermittent Hypoxia. Oncol. Rep. 32, 935–942. 10.3892/OR.2014.3298 24994549

[B96] Zou Y.-m. Hu G.-y. Zhao X.-q. Lu T. Zhu F. Yu S.-y. (2014). Hypoxia-induced Autophagy Contributes to Radioresistance *via* C-Jun-Mediated Beclin1 Expression in Lung Cancer Cells. J. Huazhong Univ. Sci. Technol. [Med. Sci. 34, 761–767. Journal of Huazhong University of Science and Technology. Medical sciences = Hua zhong ke ji da xue xue bao. Yi xue Ying De wen ban = Huazhong keji daxue xuebao. 10.1007/S11596-014-1349-2 25318890

